# Supplementary material for: Early postpartum dyslipidemia and its potential predictors during pregnancy in women with a history of gestational diabetes mellitus
Source: Lipids Health Dis. 2020 Oct 10;19:220. doi: 10.1186/s12944-020-01398-1 (PMC7547505; doi:10.1186/s12944-020-01398-1)
Supplement: Supplementary file 1 — Additional file 1. [file 12944_2020_1398_MOESM1_ESM.pdf]

Early postpartum dyslipidemia and its potential predictors during pregnancy in GDM women

by No No

Submission date: 20-Sep-2020 04:35AM (UTC+0100)

Submission ID: 133420293

File name: datacenter_paper_turnitinuk_2020-09-20_9535571.docx (121.73K)

Word count: 2766

Character count: 15366

RESEARCH

Early postpartum dyslipidemia and its potential predictors during pregnancy in women with a history of gestational diabetes mellitus

Abstract

Background: This study aimed to analyze the incidence of early postpartum dyslipidemia and its potential predictors in women with a history of gestational diabetes mellitus (GDM).

Methods: This was a retrospective study. 589 women diagnosed with GDM were enrolled and followed up at 6–12 weeks after delivery. A 75g oral glucose tolerance test (OGTT) and lipid levels were performed during mid-trimester and the early postpartum period. Participants were divided into the normal lipid group and dyslipidemia group according to postpartum lipid levels. Demographic and metabolic parameters were analyzed. Multiple logistic regression was performed to analyze the potential predictors for early postpartum dyslipidemia. A receiver operating characteristic curve (ROC) was calculated to determine the cut-off values.

Results: A total of 38.5% of the 589 women developed dyslipidemia in early postpartum and 60% of them had normal glucose metabolism. Delivery age, systolic blood pressure (SBP), glycated hemoglobin (HbA1c) and low-density lipoprotein cholesterol (LDL-C) were independent predictors of early postpartum dyslipidemia in women with a history of GDM. The cut-offs of maternal age, SBP, HbA1c values, and

LDL-C levels were 35 years, 123mmHg, 5.1%, and 3.56 mmol/L, respectively. LDL-C achieved a balanced mix of high sensitivity (63.9%) and specificity (69.2%), with the highest area under the receiver operating characteristic curve (AUC) (0.696). When LDL-C was combined with age, SBP, and HbA1c, the AUC reached to 0.733.

Conclusions: A lipid metabolism evaluation should be recommended in women with a history of GDM after delivery, particularly those with a maternal age > 35 years, SBP > 123 mmHg before labor, HbA1c value > 5.1%, or LDL-C levels > 3.56 mmol/L in the second trimester of pregnancy.

Keywords: Gestational diabetes mellitus; Predictor; Lipid, Postpartum; Cardiovascular disease

Background

Cardiovascular disease (CVD) is currently the leading cause of mortality. CVD accounts for up to 40% of all deaths in the urban and rural populations in China [1]. There has been a trend in stagnation in cardiovascular mortality rates in young adults, especially in women, even though the overall cardiovascular mortality rate has markedly decreased over the past decades [2]. Therefore, the recommendation for screening of CVD risk to be started at the age of 20 years, and revisited every 4–6 years to prevent cardiovascular events [3, 4].

Gestational diabetes mellitus (GDM) is a common pregnancy complication that is strongly associated with adverse maternal and offspring events. Currently, the incidence

of GDM in mainland China ranges 14.8%–17.6% [5, 6]. Overweight or obesity before pregnancy is one of the leading contributors to GDM [7]. Furthermore, the maternal diet during pregnancy is not only relevant for fatty acid supply during fetal life [8], but also for development of GDM. GDM can be caused by a diet low in carbohydrates, but high in animal fat and protein, as well as an overall 'Western dietary pattern' (high intake of red meat, processed meat, refined grain products, and sweets) [7]. With rapid economic growth and urbanization, the Chinese dietary pattern has become 'Westernized', resulting in an alarming increase in obesity [9]. Notably, in Chinese traditional practices, pregnant woman should eat more eggs and meat to supplement nutrition.

Women with a history of GDM have a much higher risk of postpartum diabetes [10, 11], as well as other CVD-related risk factors, including dyslipidemia [12-15] and metabolic syndrome [16]. As a result, the incidence of CVD in women with a history of GDM is 2–3-fold higher than in those without GDM [17-20]. Dyslipidemia is a major independent modifiable risk factor of atherosclerosis. A previous study showed that the prevalence of postpartum dyslipidemia in women with GDM was 52% [13] and women with GDM had a 1.4–1.8-fold risk for dyslipidemia compared with their peers [14]. These findings indicate that postpartum dyslipidemia is also a serious health problem in women with GDM. Professional guidelines recommend that all women with GDM should have glucose metabolism examined at 4–12 weeks after delivery, but the risk of postpartum dyslipidemia has not been put on the agenda [21]. To date, there were few studies focused on the risk of postpartum dyslipidemia and the potential predictors were

seldom reported. Therefore, the present study aimed to examine the potential risk factors during pregnancy affecting abnormal postpartum lipid metabolism.

Methods

Participants

Women who were diagnosed with GDM as shown by a 75-g 2-h oral glucose tolerance test (OGTT) that was performed during 24–28 weeks of pregnancy were collected. All of the women received intensive lifestyle intervention, and insulin was used for those who failed in lifestyle intervention. Participants were followed up at 6–12 weeks after delivery. Inclusion criteria were as follows: (1) age of 18–45 years; (2) a diagnosis of GDM with a 75g 2-h OGTT during 24–28 weeks of pregnancy; (3) plasma lipid was measured at the second or third trimester; and (4) women received a 75-g 2-h OGTT and plasma lipid measurements 6–12 weeks after delivery. Exclusion criteria were as follows: (1) patients diagnosed with overt diabetes during pregnancy; (2) patients suffered from subclinical or overt hyperthyroidism/hypothyroidism; and (3) patients complicated with chronic liver and kidney diseases.

Data collection

Demographic characteristics, basic anthropometry, and glucose and lipid levels during pregnancy and after delivery were recorded. Specifically, gestational age, past medical history, a history of family diabetes, pre-pregnancy weight, weight gain during

pregnancy, systolic blood pressure (SBP) / diastolic blood pressure (DBP) before labor glycated hemoglobin (HbA1c) levels, fasting plasma glucose (FPG), 1 h plasma glucose (1 h PG) and 2 h plasma glucose (2 h PG) levels of a 75-g OGTT, total cholesterol (TC) levels, triglyceride (TG) levels, high-density lipoprotein cholesterol (HDL-C) levels, and low-density lipoprotein cholesterol (LDL-C) levels were recorded.

Definitions of GDM and dyslipidemia

The diagnosis of GDM was based on the International Association of Diabetes and Pregnancy Study Groups criteria [22] in which any of the three items following 75-g OGTT were reached: FPG levels > 5.1 mmol/L and < 7.0 mmol/L, 1 h PG levels ≥ 10.0 mmol/L, and 2 h PG levels ≥ 8.5 mmol/L and <11.1 mmol/L.

Postpartum dyslipidemia was defined in accordance with the Third Report of the National Cholesterol Education Program (NCEP) Expert Panel on Detection, Evaluation, and Treatment of High Blood Cholesterol in Adults (Adult Treatment Panel III) final report (NCEP-ATP III) [23] as follows: TC levels ≥ 6.22 mmol/L, TG levels ≥ 2.26 mmol/L, LDL-C levels ≥ 4.14 mmol/L, and HDL-C levels ≤1.04 mmol/L.

World Health Organization 1999 criteria [24] were used to assess postpartum glucose metabolism of the subjects. Diabetes was diagnosed when FPG levels were ≥ 7.0 mmol/L, 2 h PG levels were ≥ 11.1 mmol/L, or random venous blood glucose levels were ≥ 11.1 mmol/L. Subjects without typical symptoms of diabetes were tested again on the following day. Impaired fasting glucose (IFG) was diagnosed as FPG levels

≥ 6.1 mmol/L and < 7.0 mmol/L and 2 h PG levels < 7.8 mmol/L. Impaired Glucose tolerance (IGT) was defined as FPG levels < 6.1 mmol/L and 2 h PG levels ≥ 7.8 mmol/L and <11.1 mmol/L.

Statistical analysis

Statistical analysis was carried out using SPSS version 22.0 software (IBM Corp, Armonk, NY, USA). Nonnormally distributed variables are presented as medians with interquartile ranges, and categorical data are expressed as percentages. Data were compared by the unpaired t-test or Mann–Whitney U test where appropriate. Categorical variables were compared using the chi-square test. Logistic regression models were used to assess the potential predictors and then adjusted for 1 h PG, TC, TG, HDL-C, and the TG/HDL-C ratio during pregnancy. The receiver operating characteristic (ROC) curve was performed to determine the cut-off values of postpartum dyslipidemia in GDM women. The overall predictability of predictors was assessed using the AUC. *P*<0.05 was considered statistically significant.

Results

A total of 589 pregnant women with GDM were enrolled and finished their postpartum visit in this study. A total of 227 (38.5%) of these women were diagnosed with dyslipidemia and 209 (35.5%, 32.4% with prediabetes and 3.1% with diabetes) were diagnosed with abnormal glucose tolerance at 6–12 weeks after delivery. A total of 23.1%

of participants had dyslipidemia with normal glucose tolerance, which accounted for up to 60% of dyslipidemia. A total of 15.49% (13.6% with prediabetes and 1.89% with diabetes) of participants had both postpartum glucose intolerance and dyslipidemia (Fig. 1a). Of these, 195 (33.1%) had abnormal TC levels, 33 (5.6%) had abnormal TG levels, 15 (2.5%) had abnormal HDL-C levels, and 127 (21.6%) had abnormal LDL-C levels. A total of 42.3% of the participants presented with only one type of dyslipidemia (Fig. 1b). Women with dyslipidemia had an older delivery age, higher levels of SBP, HbA1c, 1 h PG, TC, TG, and LDL-C, a higher TG/HDL-C ratio during pregnancy, higher postpartum glucose levels, and a higher incidence of postpartum glucose intolerance compared with women with normal postpartum lipids (Table 1).

Logistic regression analysis showed that age and SBP, levels of 1 h PG and HbA1c, the lipid profile (TC, TG, HDL-C, and LDL-C), and the TG/HDL-C ratio during pregnancy were significantly associated with postpartum lipid outcome. The odds ratios (ORs) for these variables ranged 1.047–2.551. Multivariate logistic regression analysis further showed that age (OR = 1.06, 95% confidence interval [CI]: 1.014–1.109, *P* = 0.11), SBP (OR = 1.022, 95% CI: 1.006–1.038, *P* = 0.006), HbA1c (OR = 1.897, 95% CI: 1.119–3.215, *P* = 0.017), and LDL-C (OR = 3.671, 95% CI: 1.386–9.724, *P* = 0.009) were independent predictors of abnormal postpartum lipid metabolism. ROC curves were used to predict dyslipidemia. Sensitivity in the prediction of incident dyslipidemia of each predictor varied from 47.1% (age) to 63.9% (LDL-C), and specificity decreased

from 69.6% (SBP) to 57.4% (HbA1c) across these categories. The cut-offs of age, SBP, HbA1c values, and LDL-C levels were 35 years, 123 mmHg, 5.1%, and 3.56 mmol/L, respectively. The AUCs ranged 0.56–0.696 (Table 2).

Generally, LDL-C achieved a balanced mix of high sensitivity and specificity, with the highest area under the AUC (0.696). To improve the overall predictability, the AUC was up to 0.733 when all of the independent predictors were combined (Figure 2).

Study strength and limitations

This study found that age, SBP before labor, HbA1c and LDL-C in the second trimester of pregnancy were the potential predictors in women with history of GDM, and also has a few limitations. First, the results were based on a fairly short follow-up period and all participants were from a single center. Second, a retrospective study may result in selective bias and incomplete clinical data. Finally, some characteristics of the subjects were lacking, such as breastfeeding, diet, and physical activity, which might affect glucolipid metabolism during pregnancy and postpartum. Therefore, a long-term follow-up and large-scale multicenter study for validation of our results should be performed in the future.

Discussion

We for the first time analyzed the prevalence of early postpartum dyslipidemia among Chinese GDM women. This study showed that 38.5% of women aged 18–45 years

who had a history of GDM developed dyslipidemia at 6–12 weeks postpartum. Approximately 40% of the women presented with only one type of dyslipidemia. Age, SBP before labor, HbA1c values, and LDL-C levels at 24–28 weeks' gestation were significant independent predictors of early postpartum dyslipidemia. Among them, the cut-offs were 35 years, 123 mmHg, 5.1%, and 3.56 mmol/L, respectively. The overall predictability (AUC) of LDL-C was 0.696. When LDL-C was combined with age, SBP, and HbA1c, the AUC reached 0.733. The present findings indicated that lipid levels during pregnancy were associated with an increased risk of dyslipidemia after delivery. This finding is consistent with two other studies that also showed a relationship between hypertriglyceridemia in pregnancy and at 6–12 months postpartum [25,26].

In the current study, the incidence of early postpartum dyslipidemia and hyperglycemia were 38.5% and 35.5%, respectively. The proportion of the different types of dyslipidemia in the current study is similar to that in another multi-ethnic study, which showed an overall prevalence of postpartum dyslipidemia of 52% at 6 weeks postpartum [13]. Moreover, 23.1% of participants had dyslipidemia, but normal postpartum glucose tolerance. This finding suggested that approximately one in four women with a history of GDM were mistakenly considered as "normal" if only postpartum glucose metabolism was measured. Furthermore, nearly one in six women had both postpartum abnormal glucose tolerance and an abnormal lipid profile, which indicated a high risk of CVD among these young mothers. Previous studies have shown that full adherence to the ATP III Primary Prevention Guidelines would prevent

20,000 myocardial infarctions and 10,000 deaths from coronary heart disease per year in adults [27]. Additionally, 1 mmol/L reduction in LDL-C levels could prevent 11 per 1000 major vascular events over 5 years for individuals with a 5-year risk of major vascular events < 10% [28]. Notably, more preventive efforts need to be taken for young patients with multiple risk factors for CVD who would benefit most from early cardioprotective interventions [29]. Accordingly, screening and management of dyslipidemia among these young mothers with a history of GDM at early postpartum could also be beneficial for preventing long-term CVD.

However, women with GDM have poor compliance with postpartum evaluation and the rate of postpartum review is low [30,31]. Therefore, this study examined predictors of dyslipidemia during pregnancy in women at early postpartum to raise awareness for management of early postpartum dyslipidemia. In line with previous studies [32,33], the present study showed that age and SBP were independent risk factors for postpartum dyslipidemia. Age may be a predisposing factor for dyslipidemia owing to TC, TG, and other lipoprotein levels increasing with aging [34, 35]. Remarkably, aging is associated with insulin resistance and reduced pancreatic β-cell reserve, which appears to cause exacerbated adipose tissue lipolysis [36]. Hypertensive disorders of pregnancy are also recognized as a risk of an adverse lipid profile after pregnancy [32]. As a result, guidelines recommended lipid screening for all women with a history of hypertensive disorders of pregnancy [37]. Moreover, the current study showed that HbA1c values and LDL-C levels at 24–28 weeks' gestation were independent risk

factors, and the level of LDL-C was the most important predictor of dyslipidemia. Patients with LDL-C levels > 3.56 mmol/L had relatively balanced sensitivity (63.9%) and specificity (69.2%) and had the best AUC (0.696). However, the precise mechanisms involved remain to be determined.

Management of glucose and lipids during pregnancy may provide a chance to reduce development of dyslipidemia postpartum. The ORs for age, SBP, HbA1c, and LDL-C were 1.06, 1.022, 1.897, and 3.671, respectively, in the present study. Therefore, LDL-C levels during pregnancy are the most relevant to onset of dyslipidemia after delivery, followed by HbA1c. Plasma LDL-C levels can be measured directly or calculated using the Friedewald formula as follows: LDL-C=TC−HDL-C−(TG/2.2) in mmol/L. The outcome is the same in the absence of high TG levels [38]. LDL-C induces apoptosis and decreases proliferation and maximal glucose-stimulated insulin secretion in murine and human β-cells [39]. Multiple stepwise regression analysis also showed that LDL-C and HbA1c were independent risk factors for the development of insulin resistance after delivery in Chinese women with a history of GDM [40]. Therefore, optimal levels of glucose and lipids during pregnancy might reduce insulin resistance and improve pancreatic β-cell function to decrease postpartum dyslipidemia. Maternal dyslipidemia and/or obesity affects obesity and metabolic diseases in the offspring, including changes in cardiac geometry and function [41,42]. Hyperlipidemia induces a proinflammatory cascade, which can regulate placental nutrient transporters and affect placental development and function, fatty acid composition, oxidative stress,

inflammatory stress, and adaptive immunity [43]. Obesity is implicated in increased levels of placental inflammatory markers and lipid esterification, and altered levels of maternal adipokines. This may play a role in long-term insulin resistance of offspring [44]. Additionally, dyslipidemia and obesity reduce n-3 long-chain polyunsaturated fatty acid in tissue, which is a type of fatty acid and signaling molecule acting on intracellular sensing systems to alter embryonic and fetal development. This results in long-term effects on the offspring [45]. More data are required to further verify our hypothesis in future.

Conclusion

In summary, this study shows a high prevalence of dyslipidemia in women with a history of GDM in the early postpartum period and suggests that postpartum lipid screening might be warranted. Moreover, maternal age, SBP, HbA1c, and LDL-C during pregnancy appear to be independent risk factors for developing postpartum dyslipidemia. This finding suggests that patients with GDM need more intensive treatment and optimal management of glucose, lipids, and blood pressure during pregnancy, which may be beneficial for postpartum metabolism.

Early postpartum dyslipidemia and its potential predictors during pregnancy in GDM women

ORIGINALITY REPORT

67%	%	%	%
SIMILARITY INDEX	INTERNET SOURCES	PUBLICATIONS	STUDENT PAPERS

PRIMARY SOURCES

1	www.researchsquare.com<br>Internet Source	58%
2	Ling Pei, Huangmeng Xiao, Fenghua Lai, Zeting Li, Zhuyu Li, Shufan Yue, Haitian Chen, Yanbin Li, Xiaopei Cao. "Postpartum dyslipidemia and the potential predictors in women with history of gestational diabetes mellitus", Research Square, 2020<br>Publication	1%
3	H. David McIntyre, Patrick Catalano, Cuilin Zhang, Gernot Desoye, Elisabeth R. Mathiesen, Peter Damm. "Gestational diabetes mellitus", Nature Reviews Disease Primers, 2019<br>Publication	1%
4	onlinelibrary.wiley.com<br>Internet Source	1%
5	www.dovepress.com<br>Internet Source	1%
	eprints.gla.ac.uk	

6	Internet Source	1%
7	"Gestational Diabetes During and After Pregnancy", Springer Science and Business Media LLC, 2010<br>Publication	1%
8	www.nature.com<br>Internet Source	1%
9	Submitted to El Camino Community College<br>Student Paper	<1%
10	hdl.handle.net<br>Internet Source	<1%
11	Miriam Sharpe. "Micronized Fenofibrate in Dyslipidemia", American Journal of Cardiovascular Drugs, 2002<br>Publication	<1%
12	Marco C DeRuiter. "Maternal transmission of risk for atherosclerosis", Current Opinion in Lipidology, 08/2008<br>Publication	<1%
13	worldwidescience.org<br>Internet Source	<1%
14	Geng Song, Chen Wang, Hui-Xia Yang. "Diabetes Management beyond Pregnancy", Chinese Medical Journal, 2017<br>Publication	<1%

15	Mingwei Chen, Datong Deng, Zhaohui Fang, Ming Xu, Honglin Hu, Li Luo, Youmin Wang. "Fenofibrate increases serum vaspin by upregulating its expression in adipose tissue", Endocrine, 2013<br>Publication	<1%
16	"Abstracts", Diabetologia, 1995<br>Publication	<1%

Exclude quotes	Off	Exclude matches	Off
Exclude bibliography	On		
