# Supplementary material for: Early postpartum dyslipidemia and its potential predictors during pregnancy in women with a history of gestational diabetes mellitus
Source: Lipids Health Dis. 2020 Oct 10;19:220. doi: 10.1186/s12944-020-01398-1 (PMC7547505; doi:10.1186/s12944-020-01398-1)
Supplement: Supplementary file 2 — Additional file 2. [file 12944_2020_1398_MOESM2_ESM.pdf]

理文编辑

Certificate of Editing

Edited provisional title

Early postpartum dyslipidemia and its potential predictors during pregnancy in women with a history of gestational diabetes mellitus

Client name and institution

Ling Pei, Department of Endocrinology, First Affiliated Hospital, Sun Yat-sen University

Date Completed	Identification code
2020-09-17	88273

Certificate issued by
Koji Yamashita
Managing Director and CEO

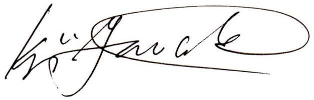

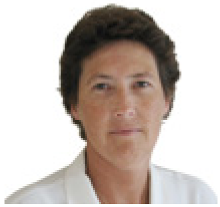

Expert Editor: Ellen Knapp
2000 PhD Developmental Medicine/Biology, Pediatrics
University of Auckland
Medical Physiology, Paediatrics and Reproductive Medicine, Biochemistry and Cell Biology

www.liwenbianji.cn
While this certificate confirms the authors have used Edanz's editing services, we cannot guarantee that additional changes have not been made after our edits.
